# Turner Syndrome and Gender Incongruence: Considerations for Gender Affirming Hormonal Therapy

**DOI:** 10.1155/crie/6687002

**Published:** 2025-02-23

**Authors:** Valerie Urban, Kanthi Bangalore-Krishna

**Affiliations:** ^1^Penn State College of Medicine, Hershey, Pennsylvania, USA; ^2^Department of Pediatrics, UPMC Children's Hospital of Pittsburgh, Pittsburgh, Pennsylvania, USA

**Keywords:** affirming therapy, gender incongruence, testosterone, Turner syndrome

## Abstract

Estrogen and growth hormone have been well established in the management of patients with Turner syndrome (TS) to improve linear growth, body composition, lipid profile, and bone mineral density. The use of testosterone therapy, however, has not been well studied in patients with TS. Furthermore, there is only one other known case report of an adult patient with TS seeking masculinizing therapy. We present an adolescent with mosaicism 45X/46XX who successfully underwent masculinizing therapy and is thriving as a young adult transgender male. This case highlights the benefits of gender affirming care despite the challenges of treatment in a population not previously described.

## 1. Introduction

Patients with Turner syndrome (TS) have a wide range of associated comorbidities requiring multidisciplinary care including cardiology, endocrinology, psychiatry, nephrology, orthopedics, otolaryngology, and dermatology [1]. Specifically, many (80%–90%) but not all patients with TS have primary ovarian insufficiency and require estrogen replacement therapy starting at 11–13 years of age through adulthood to initiate and maintain female pubertal changes and improve bone health and metabolic parameters like HDL levels [2]. Additionally, initiating growth hormone (GH) in childhood improves final adult—height, body composition, and lipid profile [1]. There is also some data supporting testosterone supplementation in improving cardiovascular disease (CVD) risk factors, as TS is often associated with obesity [3]. Each patient's specific genetics, comorbidities, and long-term goals must be considered in the overall management, especially in cases of mosaicism. We present an individual with TS assigned female at birth, mosaic, 45X/46XX, with gender incongruence, expressing desire to undergo masculinizing therapy. This case emphasizes the importance of personalized treatment plans for patients with TS and gender dysphoria, which has only been described thus far in one other older transgender male patient with TS [4].

## 2. Case

**Figure 1:**
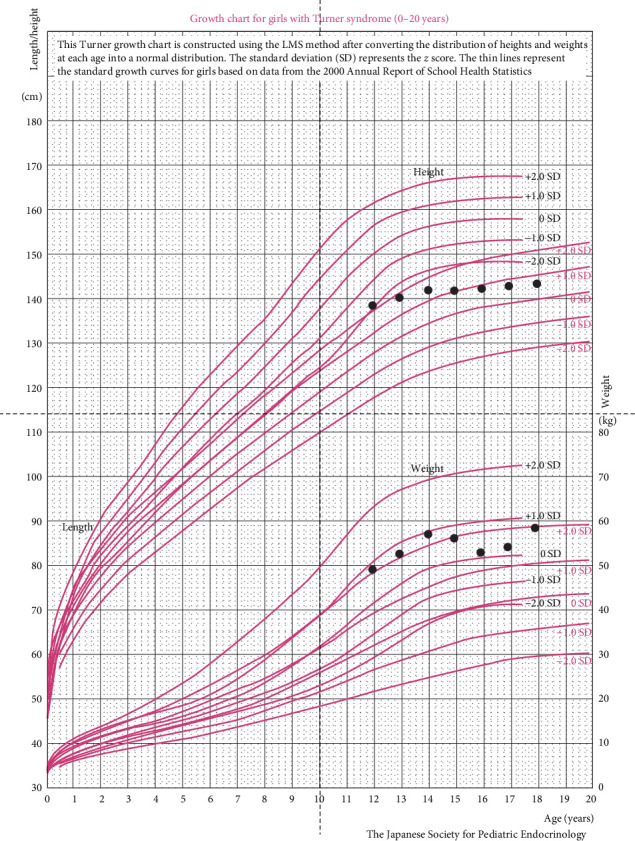
Growth chart as plotted on the Turner syndrome growth curves. *Source:* T. Isojima, S. Yokoya, J. lto, et al. Clin. Pediatr. Endocrinol. 19, (2010): 69–82.

Patient is an adopted, Asian-origin, assigned female at birth individual who was diagnosed with TS at age 12 via genetic testing (45X/46XX) to investigate short stature. At that time, the patient was noted to have had menarche at age 11 3/12 years and menstrual periods lasting approximately 18 days up to two times per month over the past year. The patient was found to have mosaicism with monosomy X in 8 of 50 cells with no Y-chromosome material detected. This led to an initial endocrine evaluation and consideration for the standard estrogen replacement and GH therapy. The patient, however, had already undergone spontaneous menses with Tanner stage IV pubic hair, stage V breast, and bone age of 13 years and 9 months at the time of initial presentation (achieved >96% of their growth upon evaluation). Physical exam was also notable for low hairline on posterior neck, height at <3rd percentile and BMI at >95th percentile, with no target height range since the patient was adopted (Figure 1). The patient had no underlying cardiac defects associated with TS like bicuspid aortic valve or coarctation of aorta. The patient was started by an adolescent gynecologist on an oral contraceptive pill and had well-regulated menses on these medications. GH was not recommended due to limited potential benefit. The patient did have a history of sensorineural hearing loss at age 7 years requiring a hearing aid with MRI showing slight prominence of posterior fossa CSF space consistent with either mega cisterna magna variant or Dandy–Walker variant. The patient was in otherwise good health with no cardiovascular or renal concerns, normal blood pressure, normal lipid panel, liver enzymes, thyroid studies, and gonadotropin levels (Table 1). The only abnormal hormonal studies at age 12 were a mildly elevated DHEAS (220 mcg/dL ref range 35–192) and free testosterone (3.9 pg/mL, ref. range 0.7–3.6). The patient lived with their adoptive parents and adoptive sister, with both children adopted from different cities in China.

By age 14, the patient began discussing identification with masculinity to express identification as a male individual, desire to have a broader, flatter chest and phallus, and distress regarding uncertainty about gender identity for at least 3 years. The patient wished to live as male and transition to a new, more masculine name. The patient was counseled regarding the possibility of initiating puberty blockers and then discussing gender affirming hormonal therapy if desired. The patient continued to meet with a mental health provider who attested to their decision and the patient remained confident in their decision to transition to male gender. We recommended that the patient meet a reproductive endocrinologist for consideration of future reproductive function and fertility options including oocyte cryopreservation and ovarian tissue cryopreservation. Puberty blockers were not initiated due to pending cryopreservation discussions; however, the patient preferred to continue on the oral contraceptive pill—FSH, LH, and AMH levels were not obtained off OCPills. Patient also did not want to switch to progesterone-only medication which would be an appropriate choice for menstrual suppression in a person expressing desire to undergo masculinizing therapy. After consultation with the reproductive endocrinologist, the patient ultimately decided that he did not want biological children and did not want to pursue any of the currently available options for fertility preservation despite the knowledge that individuals have been able to get pregnant and/or use their gametes after years on testosterone [5]. Testosterone therapy was initiated at close to 16 years of age (Injectable testosterone enanthate, 25 mg/m^2^/2 weeks) and escalated per the Endocrine Society Guidelines. There were no absolute contraindications to initiating this treatment, but we recommended that he follow with a cardiologist considering the impact of testosterone therapy on CVD risk is not well studied in patients with TS. The patient had some risk factors for CVD with a BMI > 90th percentile, but his blood pressure was overall stable (Table 1) and his lipid panel was within normal limits prior to initiating testosterone (Table 1). ECHO, EKG, and cardiac MRI were normal at annual cardiology follow-up in the first 2 years after starting testosterone. Age-appropriate contraceptive counseling was discussed at each visit, in particular emphasizing the need for strategies to prevent sexually transmitted infections. We also discussed that gender affirming hormonal therapy is not a method of hormonal contraception and that unintended pregnancies may occur despite amenorrhea.

The patient underwent “top surgery” 1.5 years year after initiating testosterone and had started to experience a deepening of his voice with more facial hair, axillary hair, pubic hair, and abdominal hair. The patient continued to follow regularly with both psychiatry and his therapist to provide ongoing gender counseling.

By age 18, 2 years after initiating testosterone, the patient had an increase in LDL (as is expected with lower estradiol levels) but total cholesterol and TG were within normal limits (Table 1). Blood pressure remained stable for age and affirmed sex (Table 1).

The patient developed Hashimoto's thyroiditis at age 18 with elevated TSH, low normal free T4, and positive thyroid peroxidase (TPO) antibodies, and levothyroxine therapy was initiated. The patient and his family were satisfied with the outcomes thus far of his transition with the gender affirming hormone therapy, and he expressed a desire to pursue phalloplasty in the future. He performed well throughout school, engaged in a supportive relationship with a partner, and enrolled in college with a plan for close follow-up with endocrinology and gender and psychiatric counselors.

## 3. Discussion

There is minimal known data regarding the safety of testosterone as gender affirming therapy in patients with TS. There is only one other known case report of a transgender male with TS, who elected to undergo modified masculinization therapy with nonaromatizing androgens [4]. Therefore, it is important to have an in-depth discussion regarding the risks versus benefits based on each patient's unique case and their ultimate goals of care. Although the FDA does mandate a possible increase in cardiovascular events risk with testosterone therapy, other studies demonstrate equivocal evidence. Particularly in regard to TS, one random double-blind placebo crossover pilot study of 14 TS women aged 17–27 treated with estrogen/progestin and 1.5 mg oral methyl testosterone or placebo for 1 year showed testosterone significantly reduced total cholesterol, fat mass, and triglycerides [3]. Furthermore, a case-control study showed patients with hypogonadism treated with testosterone had a rate ratio of thromboembolism of 1.52 (0.94–2.46) compared to a rate ratio of 1.88 (1.02–3.45) in those without hypogonadism [6]. Thus, although testosterone is not well studied in patients with TS, there is some evidence indicating that it may be safely utilized and perhaps even provide some cardiovascular benefit. One study showed that transmasculine persons receiving testosterone may be at higher risk for myocardial infarction [7]. Nevertheless, discussing the potential risks thoroughly with each patient initiating gender affirming care is imperative prior to starting treatment. Furthermore, treatment of gender dysphoria requires multidisciplinary care for both physical and psychosocial support.

Ultimately, our patient was pleased with the outcome of their transition and went on to find success both academically and socially. He established a meaningful relationship with a supportive partner and enrolled in college. Despite the unknown risks of testosterone therapy for this patient with TS, appropriate support and gender affirming care have been shown to improve health outcomes for patients with gender incongruence. Ongoing mental health support is an important consideration, as symptoms of moderate to severe depression and anxiety have been seen in over 50% of transgender individuals, although this is thought to reflect societal stigma rather than any underlying vulnerability [8]. Encouragingly, one study of over 600 transgender individuals showed family social support was associated with resilience and correlated with symptoms of anxiety and depression (*r* = −0.31 and −0.37, respectively, *p*  < 0.01) [8]. Our patient safely transitioned to male with gender affirming hormone therapy, top surgery, interdisciplinary care, and the support of his family. He is a unique example of how caring for a specific patient with TS and gender dysphoria requires adaptation based on the goals and health of the individual patient.

## Figures and Tables

**Table 1:** Laboratory data before and after starting affirming therapy with testosterone.

Lab measurement/clinical parameter	Reference range	Baseline prior to starting testosterone	One year after starting testosterone	Two years after starting testosterone
RBC (M/μL)	3.8–5.34 (natal female)	5.05	5.5	5.81
Hgb (gm/dL)	11.5–15.5 (natal female)	14.9	16.6	16.9
Hct (%)	34.5–47.0	43	48.1	49.5
LDL-C (mg/dL)	<100	85	—	111
HDL-C (mg/dL)	low risk >60 high risk <40	53	—	45
FSH (mIU/mL)	14–17 years 0.64–10.98	5.56	5.10	—
LH (mIU/mL)	12–14 years 0.04–10.80 15–17years 0.97–14.70	2.28	3.92	—
DHEAS (ug/dL)	102–356	385	—	—
Free testosterone (pg/mL)	0.8–2.4	3.3	—	—
Testosterone (ng/dL)	350–970	—	320 (testosterone dose was IM 50 mg/m^2^/2 weeks	798 (testosterone dose was IM 75 mg/m^2^/2 weeks)
Systolic BP percentile (for age and natal sex)	—	66	64	73
Diastolic BP percentile (for age and natal sex)	—	76	77	73

*Note:* Baseline lab studies prior to initiating testosterone therapy at 16 years of age compared to labs at 1- and 2-year follow-up after starting testosterone. (Baseline LDL-C, HDL-C, FSH, and LH at age ~14 years. BP, blood pressure percentile for age and natal sex. (Baseline CBC at age 12 years. Baseline BP at age 15 9/12 years right before starting affirming therapy).

## Data Availability

The deidentified patient data used to support the findings of this study have been made available after patient and parental consent, and any additional data required may be requested from the corresponding author at bangalorekrishnak2@upmc.edu.
